# Why is didactic transposition in disaster education needed by prospective elementary school teachers?

**DOI:** 10.1016/j.heliyon.2023.e15413

**Published:** 2023-04-18

**Authors:** Eddy Noviana, Almasdi Syahza, Zetra Hainul Putra, Hadriana, Yustina, Sri Erlinda, Desfi Rahmi Putri, M. Arli Rusandi, Dominikus David Biondi Situmorang

**Affiliations:** a Doctorate Program of Education, Universitas Riau, Indonesia; b LPPMP, Universitas Riau, Indonesia; c Department of Guidance and Counseling, Universitas Riau, Indonesia; d Department of Guidance and Counseling, Atma Jaya Catholic University of Indonesia, Indonesia

**Keywords:** Disaster mitigation education, Disaster knowledge, Disaster mitigation knowledge, Prospective elementary school teachers

## Abstract

Disaster risk reduction is a significant focus on sustainable development. One way to reduce disaster risk is through disaster education. Through disaster education, disaster knowledge and disaster mitigation knowledge will be obtained. This research is a preliminary study of didactic transposition in disaster education. The method used in this study is the SLR approach and bibliometric analysis. The research findings indicate four forms of connectedness, classified based on the main keyword, disaster knowledge. The four linkages are described as (a) co-occurrence network analysis; (b) word cloud analysis; (c) word tree maps analysis; and (d) network visualization analysis. Subsequently, the findings of the four connectedness are grouped into four clusters. The first cluster is disaster risk reduction, the second cluster is knowledge, the third cluster is disaster mitigation, and the fourth cluster is disaster knowledge. The four connectedness and four clusters will be used as recommendations for future research on the design and development of didactic transpositions in disaster education for prospective elementary school teachers.

## 1 Introduction

Indonesia has a region with its uniqueness and characteristics in the world. Indonesia has the world's largest island and longest coastline, with over 17,000 islands and over 80,000 km of coastline. In terms of biodiversity, Indonesia ranks third after Brasilia and Colombia. However, behind these advantages, Indonesia has the most active volcanoes globally that meet tectonic plates that can cause disasters. The disasters are volcanic eruptions, earthquakes, and tsunamis. The disaster occurred because Indonesia is a region where tectonic plates confluence, namely Eurasia, Indo-Australia, the Philippines, and the Pacific [1]. It shows that Indonesia is an area traversed by the ring of fire, resulting in most areas in Indonesia being areas with high disaster risk. Disaster risk in this region of Indonesia can be seen from 34 provinces, 19 (55.88%) provinces are in the high disaster risk category, and 15 (44.12%) provinces are in the medium disaster risk category (Table 1).

**Table 1:** Disaster risk index in Indonesia.

No	Provinces	Risk Score	Risk Class	No	Provinces	Risk Score	Risk Class
1.	West Sulawesi	166.49	High	18.	South Kalimantan	144.81	High
2.	Bengkulu	162.00	High	19.	West Papua	144.05	High
3.	Bangka Belitung Island	161.54	High	20.	Special Region of Yogyakarta	140.92	Medium
4.	Maluku	160.35	High	21.	East Nusa Tenggara	140.89	Medium
5.	South Sulawesi	159.49	High	22.	North Sulawesi	139.47	Medium
6.	South-East Sulawesi	157.72	High	23.	South Sumatera	139.24	Medium
7.	Banten	154.87	High	24.	Jambi	138.64	Medium
8.	East Kalimantan	154.02	High	25.	West Kalimantan	138.49	Medium
9.	North Kalimantan	153.62	High	26.	East Java	134.39	Medium
10.	Aceh	153.58	High	27.	Central Java	132.99	Medium
11.	West Sumatera	149.53	High	28.	Central Kalimantan	132.70	Medium
12.	Riau	147.27	High	29.	Bali	129.43	Medium
13.	Lampung	146.78	High	30.	West Nusa Tenggara	128.05	Medium
14.	West Java	145.81	High	31.	Gorontalo	126.64	Medium
15.	North Maluku	145.63	High	32.	Papua	122.90	Medium
16.	North Sumatera	145.18	High	33.	Riau Island	116.40	Medium
17.	Central Sulawesi	144.96	High	34.	Jakarta	64.02	Medium

Source: BNPB, 2020 [1].

Based on Table 1, it can be seen that most of Indonesia's territory is at a high level of disaster risk. It can be stated that Indonesia's territory is a disaster-prone zone. It is proven by many provinces in Indonesia that have disaster risk category levels. West Sulawesi, for example, has the highest disaster risk category with a risk score of 166.49, and DKI Jakarta is a province with a moderate risk category with a risk score of 64.02. It proves that Indonesia is legitimately categorized as a disaster-prone area. In line with this, data released by the National Disaster Management Agency (BNPB) from 2021 to April 2022 recorded 1125 natural disasters that hit Indonesia and resulted in fatalities and damage to buildings [1,2]. Then, based on the disaster index conducted by BNPB, the tsunami disaster is the disaster with the highest risk in Indonesia, with a score of 9.7 points, followed by an earthquake with a score of 8.9 points, and a flood disaster with a score of 8.1 points. The disaster risk score that has been described determines the highest disaster risk index in Indonesia [1]. Based on the disaster risk index, tsunamis, earthquakes, and floods often occur in Indonesia.

According to geoportal data in Indonesia (https://gis.bnpb.go.id/) issued by BNPB from 2019 to 2022, it provides information on the average numbers of incidents based on the type of disaster, which are (a) flood at 1407 incidents; (b) landslides at 932 incidents; (c) tornado at 693 incidents; (d) extreme weather with 661 incidents; (e) forest and land fires at 544 incidents; (f) tidal wave and abrasion at 45 incidents; (g) drought at 42 incidents; (h) earthquakes at 25 incidents; and (i) volcanic eruptions at five incidents. Therefore, Indonesia's average number of disaster incidents during the last four years was 435, with an average of 780 incidents per year. The disaster that happened the most was the flood disaster, while the disaster that happened the least was the volcanic eruption.

A disaster will undoubtedly result in disaster impacts such as the impact of victims who died, the impact of missing victims, the impact of victims who were injured, and the impact of victims who suffered and were displaced. According to information provided by BNPB in 2022, the average impact of disasters in Indonesia from 2019 to 2022 included the following statistics in which (a) the impact of fatalities was 611; (b) the impact of victims who went missing was 72; (c) the impact of victims who suffered and were displaced was 6,667,931; and (d) the impact of injuries was 6921 (https://gis.bnpb.go.id/). Based on the information, it can be shown that 6,675,534 people were affected by catastrophes in Indonesia on average over the past four years, with as many as 1,668,883 people per year. Disasters also result in destruction, including damage to homes, facilities, workplaces, and bridges and their negative effects. The average breakdowns of the disaster's damage indicate (a) the damage to homes at 82,728; (b) the damage to facilities at 2267; (c) the damage to offices at 303; and (d) the damage to bridges at 417. Thus, there were 21,429 disaster-related losses over the previous four years, for an average annual loss of 88,796.

According to the previous description, Indonesia is a geographically vulnerable country and region. Due to the catastrophic impact and damage produced by the disaster, it can be inferred from the data on the impact of disasters and the damage they cause that Indonesia continues to experience a high level of disaster-related impact and damage. As a result, it is important to be aware of disaster mitigation in the community to reduce the impact of catastrophes and the damage they create.

People who live in disaster-prone areas should prepare for, anticipate, and adapt to disasters. Preparedness, anticipation, and adaptation are parts of disaster mitigation knowledge. With that knowledge, we hope it can increase the community's awareness and guidance about disaster management as early as possible [[3], [4], [5]]. Therefore, through knowledge of disaster mitigation, knowledge, and public awareness will be built to overcome and minimize the impact of disasters to occur. In addition, disaster education has been implemented in many disaster-prone countries, such as the Philippines, India, Indonesia, and Fiji Islands. Japan, for example, has implemented intensive disaster education since the primary school level [6,7], while in Fiji Island and the Philippines, the education is reflected through arts and literature. Looking at the examples, Indonesia is expected to have a similar degree of disaster education implementation. However, one issue Indonesian teachers face is that they are not well-equipped with the relevant skill and knowledge for disaster education [[8], [9], [10]]. Therefore, education practitioners need to fill this gap with adequate knowledge related to disaster education, which can be used in their classrooms.

Building disaster awareness is vital for countries with high disaster risk, especially in Indonesia [1,2,[11], [12], [13]]. One of which is disaster education [14,15]. The essence of disaster education is expected to provide knowledge to minimize the impact and damage caused by disasters [[16], [17], [18], [19]]. Thus, it can effectively build awareness in dealing with disasters [20,21].

Research on disaster education obtained from theoretical and empirical literature studies regarding the urgency of disaster mitigation knowledge for prospective teachers is essential. Research on the impact of education on disaster vulnerability in the Southeast Asian region states that through education and learning, individuals acquire knowledge, abilities, skills, and perceptions that enable them to prepare themselves effectively for and cope with disasters [22]. Subsequently, the research identifies that the relationship between disaster prevention awareness and concern for disaster preparedness is very relevant, so disaster preparedness education needs to be recommended to prepare teachers to know about disaster mitigation [23]. Based on other research on disaster resilience learning opportunities in the Australian curriculum, schools are essential for students to learn disaster resilience [24]. Therefore, the teacher's knowledge of disaster is a success factor in implementing disaster preparedness learning.

All research above is a study on disaster education that is carried out using an empirical and theoretical approach to state the conclusions from the results of the research that has been conducted. Only a few have surveyed the existing literature to explain disaster education, disaster knowledge, and disaster mitigation knowledge. Thus, this research will describe the relationship between disaster knowledge and disaster mitigation knowledge as the primary source of content in disaster education for prospective elementary school teachers through a systematic literature review (SLR) approach. SLR is a research approach that summarizes the results of preliminary research to present facts that are more comprehensive and balanced [25,26] used for scientific research that focuses on specific questions in identifying, selecting, assessing, and summarizing findings from similar studies [[27], [28], [29]], which in this case are about disaster education, disaster knowledge, and disaster mitigation knowledge.

In addition, this research also uses bibliometric network analysis to establish connectivity and identify the general focus of the articles used as reference sources in this research. Network analysis facilitates the identification of the most significant articles while extracting their output and determining their grouping [30]. Subsequently, it describes the significant results of the research direction and the main themes that will attract the scientific community's attention. Therefore, we utilize bibliometric network analysis, which describes the objectives of the research: to conduct investigations on disaster knowledge and disaster mitigation knowledge as well as to produce a research topic in the future, namely didactic transposition in the context of implementing disaster education for prospective elementary school teachers.

This research is a preliminary study on didactic transposition in disaster education. Didactic transposition is the competency of prospective elementary school teachers in teaching disaster content and disaster mitigation through educational processes, courses, and training. Hence it can be utilized in learning about disaster content in elementary schools. It is hoped that the disaster mitigation knowledge possessed by prospective teachers in elementary schools can be transferred back to students so that students can have disaster knowledge, disaster mitigation knowledge, and learn resilience in dealing with disasters.

## 2 Research methodology

### 2.1 Method

**Fig. 1:**
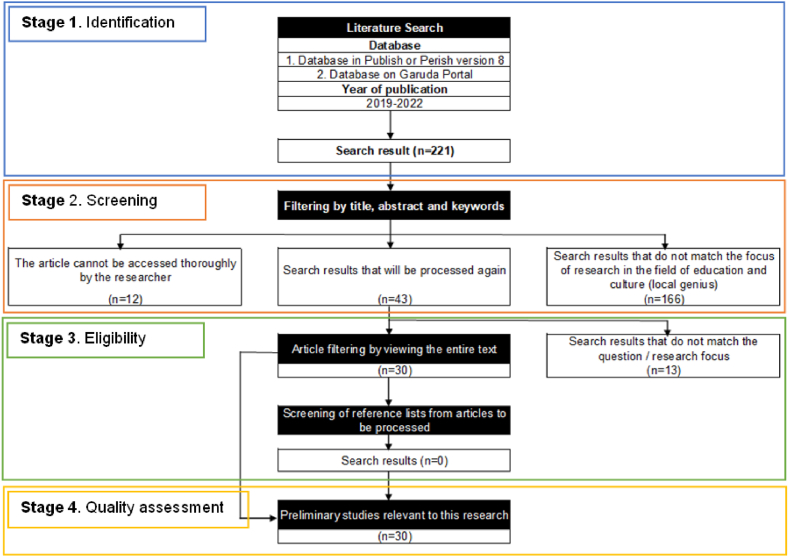
Stages of the search and selection of paper (PRISMA Flow Chart Diagram).

The research method used is a SLR. SLR is a research method that summarizes preliminary research results to present more comprehensive and balanced facts [25,26]. SLRs are scientific research that focuses on specific questions and uses a defined, explicit scientific method to identify, select, assess, and summarize findings from similar studies. In addition, this method provides a deeper understanding of the research question through an extensive database of the articles to be reviewed. The main reason for choosing SLR is to adopt an excellent literature review in the research on disaster knowledge, disaster knowledge, and disaster education, which will be used as a preliminary study of didactic transposition in disaster education in elementary schools. The pattern of this research in determining criteria and data collection uses Preferred Reporting Items for Systematic Review and Meta-Analyses (PRISMA). By adopting the PRISMA, the stages are (a) the identification; (b) the screening; (c) the eligibility; and (d) the quality assessment (Fig. 1).

### 2.2 Systematic search strategy

#### 2.2.1 Identification

The identification is the stage in the literature search from database sources. A literature search from the database is collected in two ways. The first way is to use the Publish or Perish (PoP) application version 8 for Windows to search for English-language published articles in Scopus-indexed journals. Meanwhile, the second way is to directly access the Garuda Portal database page (https://garuda.kemdikbud.go.id/journal) to search Indonesian-language publications published in journals in Indonesia (Table 2).

**Table 2:** Selection strategy.

Database	Method	Search String	Results
Scopus	Indirect, using PoP Software	KEY (disaster AND knowledge) AND LIMIT-TO (PUBYEAR, 2022) OR LIMIT-TO (PUBYEAR, 2021) OR LIMIT-TO (PUBYEAR, 2020) OR LIMIT-TO (PUBYEAR, 2019) AND (LIMIT-TO (DOCTYPE, “ar”) OR LIMIT-TO (DOCTYPE, “cp")) AND (LIMIT-TO (EXACT KEYWORD, “Knowledge")) AND (LIMIT-TO (OA, “all"))	200
Portal Garuda	Direct, with Website	Search for "*pengetahuan bencana"*, by title, from 2019 to 2022	21

The reason for using PoP is to ease the authors to track, retrieve, and analyze articles contained in the database on Scopus. PoP can also help to select articles in the Scopus database according to the quality of the articles. It refers to accessing the Scopus database via PoP and entering the API key in the preferences menu. The API key can be accessed free of charge and obtained through registration at https://dev.elsevier.com/. In addition, the database on the Garuda Portal is a database of scientific journals developed by the Ministry of Education, Culture, Research and Technology of the Republic of Indonesia, which is used as a repository for Indonesian-language journals published in Indonesia.

The next step is to search using the keywords “*disaster knowledge*” for articles published in English and "*pengetahuan bencana*" for articles published in Indonesian. Subsequently, the search for articles in both databases is limited to issues from 2019 to 2022. The literature search results indicate 221 articles, which are 200 articles published in Scopus-indexed international journals and 21 in journals published in Indonesia.

#### 2.2.2 Screening

The screening stage is applied through filtering based on the title, abstract, and keywords. Screening results indicate 43 articles of which number of articles came from (a) 12 articles that could not be accessed as a whole and (b) 166 articles that were not put in this research are the focus of research in the field of education and culture (local genius). The reason for choosing the field of education and culture is that education and culture are mutually integrated. Education constantly changes following cultural developments because education is a process of transferring cultural values [[31], [32], [33], [34], [35], [36], [37], [38]]. Education is also a process of involving someone in a culture and making them behave based on each culture [31,35,39]. Therefore, education and culture are formed into a system that can support the achievement of educational goals, in this case, disaster education. It is hoped that disaster education can provide disaster knowledge and disaster mitigation knowledge for prospective elementary school teachers as a form of didactic transposition.

#### 2.2.3 Eligibility

In this stage, manual checking is carried out by evaluating the title and abstract to ensure that all articles meet the criteria by looking at the contents of the entire text in the article. Several articles not related to the research theme were excluded from the list of 13 articles. After an eligibility check, 30 articles were obtained.

#### 2.2.4 Quality assessment

This stage is a validation process for the retrieved articles. Selected articles come from publications indexed by Scopus, Garuda Portal, or both. We selected articles registered on the Scopus website and the Garuda Portal during the specified period from 2019 to 2022. Thus, this research examined 30 articles, consisting of 17 English articles published in Scopus-indexed journals and 13 Indonesian-language articles published in journals in Indonesia.

### 2.3 Data presentation and analysis

We conducted a descriptive analysis by presenting information on the article's progress by reviewing the journal, author, and country. In addition, we grouped them through network and content analysis based on the main keywords, namely disaster knowledge, through bibliometric analysis. Bibliometric analysis was performed using *Biblioshiny for Bibliometrics* and VOSviewer software. In addition, we conducted a thematic analysis by examining the similarity of patterns across the articles.

## 3 Results

### 3.1 Descriptive analysis

In this section, fundamental facts regarding the source of the data, the result of research and analysis of disaster knowledge, classification of indigenous disaster mitigation by themes belief, and classification of disaster knowledge will be described to find the similarities between previous articles and the research in this paper. It can be seen in Table 3 as follows.

**Table 3:** Fundamental facts regarding the source of the data.

Criteria	Description	Result
Main Information	Year	2019–2022
	Documents	30
	Garuda Database (Local)	13
	Scopus Database (International)	17
	Average publication per year	7.5
	Average citations per document	11.86
	Average citations per year per document	9.77
	Average references per document	29.62
	Average references per year per document	7.77
Document	Article	24
	Conference Paper	6
Abstracting and Categories	Scopus (Q1)	7
	Scopus (Q2)	1
	Scopus (Q3)	1
	Scopus (Q4)	6
	Scopus (Non-Q)	2
	SINTA-indexed journal (Sinta 3)	2
	SINTA-indexed journal (Sinta 4)	5
	SINTA-indexed journal (Sinta 5)	1
	Local journal not indexed by SINTA	5
Authors	Authors	96
	Average authors per document	3.22
	Average authors per year per document	0.80
Author's Country	Indonesia	22
	China	2
	Philippines	1
	Philippines, Hong Kong, Canada	1
	Taiwan	1
	China & Pakistan	1
	Denmark	1
	India	1

As regards Table 3 above, there are four criteria regarding the sources of the data, main information, documents, abstracting and categories, and authors. In the main information section for 2019–2022, the highest number of articles was obtained from the Scopus database, with 17 documents. In addition, the most significant number in the document section was 24 journal articles. Subsequently, in the abstracting and categories section, most articles came from Scopus-indexed journals (Q1) with seven documents. Moreover, based on the author's side, the number of authors from the 30 articles was 96. Most of them came from Indonesia, with a total of 22 authors. It implies the basis for determining and conducting analysis because the article's research result contains disaster knowledge content and disaster mitigation knowledge as the main component of didactic transposition for prospective elementary school teachers in disaster mitigation education.

In general, research on disaster knowledge provides information that there has been an increase in respondents' knowledge, understanding, and attitudes about disaster preparedness. Increasing knowledge, understanding, attitudes, and disaster preparedness are carried out through the application of learning methods, disaster management training, outreach activities, training on the use of GPS for education, and integration into the curriculum, as well as the use of information systems and technology tools in learning for research in the field of education. As for the cultural sector, increasing knowledge, understanding, attitudes, and disaster preparedness is carried out through socialization and applying local wisdom related to the environment and nature in the community. The results of research and analysis of disaster knowledge from 30 articles can be described in Table 4.

**Table 4:** The result of research and analysis of disaster knowledge.

No	Authors	Keywords	Results
1	Friska Ayu, Ratna Ayu Ratriwardhani (2021)	fire disaster preparedness, Islamic boarding school, Santri SIGAB	Provide information on a relationship between the level of knowledge and students' attitudes towards fire disaster preparedness in Islamic boarding schools. Four parameters that can be measured to see a person's preparedness in a disaster are knowledge of potential hazards, attitudes, emergency planning, warning systems, and resource mobilization in the event of a disaster.
2	Samson CMS, Wina Erwina, Elnovani Lusiana (2021)	local knowledge; disaster mitigation; *Tatar Karang*	Provide information that other perspectives are needed in viewing disaster mitigation activities, such as local knowledge about disaster mitigation inherited from ancestors, obedience based on customs, religion, and the state, manifested in concrete actions.
3	Siti Irene Astuti Dwiningrum et al. (2021)	disaster mitigation, disaster knowledge, disaster risk	Provide information that the average level of disaster knowledge reaches 50%. From these data, it can be interpreted that schools should provide more effective socialization and training for students related to disaster knowledge because it is essential for students, and schools are essential in disaster mitigation.
4	A Hatibe, A Salam, M Ali, Gustina (2021)	knowledge of disasters, community preparedness, landslide preparedness	Providing information that disaster knowledge and community attitudes have a positive and significant impact on preparedness in dealing with landslides, based on the data, it can be seen that if disaster knowledge is not applied to concrete actions, community preparedness in dealing with landslides will not increase significantly.
5	Yixuan Zou, Laijin Shen, Shabnam Dadparvar (2022)	e-learning behaviour, students, learning performance, influence, disaster emergency knowledge	Providing information literacy, technical characteristics, and the condition of facilities have a positive and significant impact on student learning performance from adopting disaster emergency knowledge with e-learning.
6	Rusli Yusuf, Muhammad Yunus, Maimun, Iwan Fajri (2021)	environmental literacy, disaster knowledge, environmental sensitivity, clean living behaviour	Provide information that environmental literacy, disaster knowledge, and environmental sensitivity positively correlate with clean living behaviour. Environmental literacy, disaster knowledge, and environmental sensitivity encourage clean living behaviour. The government should be concerned about the elements by incorporating the concept into the school curriculum as early as possible. Environmental literacy is critical to increasing environmental sensitivity among the Acehnese people in Indonesia.
7	Eko Prasetyo, Nur Yeti Syarifah, Yuli Ernawati (2020)	training, preparedness, mountain eruption, knowledge, students	Provide information that there is an increase in preparedness knowledge in students after disaster management training.
8	Felive R.D.C.Pasaribu, Mori Agustina br Perangin-angin (2020)	attitude, earthquake, knowledge	Provide information that the knowledge possessed by high school students is suitable, but they can still not take the right attitude to deal with earthquake disasters. Therefore, it is expected to hold regular training or socialization related to this matter.
9	Riska Oktavia Siregar, Yeni Solfiah, Hukmi (2020)	knowledge, disaster management, haze	Provide information that the description of PAUD teachers' knowledge about haze disaster management is 82.71%, which is in the excellent category in meeting the indicators to be said to be good in haze disaster management.
10	Nia Maharani, I Kadek Ariesta Andika (2020)	student, school, preparedness for earthquake	Provide information that the level of student's knowledge about preparedness for natural earthquake disasters is in the excellent category with experience of earthquake disasters and counselling provided by community leaders, health workers, and others that make students more aware of the causes of earthquakes and earthquake mitigation.
11	Juli Purnama Sari, Budi Satria (2020)	mitigation, tsunami disaster, community knowledge level	Providing information that the community has a good understanding of tsunami mitigation, the community already understands what to do when a tsunami disaster occurs.
12	Ayub Esperanza, Samuel M. Simanjuntak (2020)	earthquake readiness promotion and drilling, knowledge, college student	Provide information that the level of knowledge of students' earthquake preparedness increases after being given promotions and training on earthquake preparedness.
13	Lastri Lestari, Eddy Noviana, Zetra Hainul Putra (2020)	knowledge, disaster mitigation, forest and land fires, descriptive quantitative	Provide information that the knowledge of forest fire mitigation in elementary school students is in a suitable category. The analysis results show the elaboration of four indicators before a disaster during a disaster. After a disaster, for factual, procedural, and metacognitive indicators to get the highest average value during a disaster with a suitable category, on conceptual indicators, the highest average value is when a disaster occurs, namely in the category good.
14	Nur Hasna Pratiwi, Upik Elok Endang Rasmani, Nurul Shofiatin Zuhro (2020)	disaster mitigation, disaster preparedness, early childhood, flood	Provide information on applying flood disaster preparedness activities to increase knowledge of flood disaster mitigation.
15	Yunasril, Heri Prabowo (2019)	GPS, lab sheet, digital media, earthquake	Providing information that training on the use of GPS as knowledge in mapping the early morning earthquake for teachers can increase teacher knowledge, then the location of local areas with the potential for liquefaction and severe damage can be mapped using GPS.
16	Ni Made Wiwik Astuti, I Komang Werdhiana, Unggul Wahyono (2020)	disaster risk reduction, disaster experience, curriculum, education, Central Sulawesi	Provide information that teachers are increasing in terms of disaster risk reduction knowledge, but teachers also bring information related to disasters and disaster risk reduction (disaster mitigation) in-class activities. The role of schools in reducing disaster risk also appears after a disaster occurs, both in terms of disaster mitigation topics and activities. However, due to data limitations, researchers cannot confirm whether the program has been permanently included in the school curriculum or only as a temporary activity.
17	Kristoffer Albris, Kristian Cadervall Lauta, Emmanuel Raju (2020)	disaster governance, disaster risk reduction, Europe, knowledge sharing, risk expertise, science-policy interface	Provide information that disaster management is increasingly complex in modern times. Policymakers are faced with priority decisions for disasters. One of the challenges facing the interface of science policy on disaster risk reduction in Europe is a lack of cooperation between science and policy, so knowledge-sharing activities are needed between the people behind science, policy, and society.
18	Ginbert Permejo Cuaton, Yvonne Su (2020)	disaster risk reduction, local-indigenous knowledge, disaster, indigenous people, Haiyan, Philippines	Provide information that the acquisition of knowledge and understanding of local knowledge can be a way to mediate with the surrounding environment and is one contribution to recovering, reducing effects, and recovering from natural hazards. For example, the knowledge of the traditional elders and their advice based on what was given to them based on indigenous knowledge proved helpful and contextually reliable in preparing for the typhoon Haiyan disaster.
19	Rizki Kurnia, Ahmad Fauzi (2020)	disaster management volcanoes, disaster knowledge, mount erupts	Provide information that students' knowledge of volcanic disaster mitigation is at a percentage of 61.33%, which is categorized as very poor. Following the curriculum demand that learning materials should be implemented based on the potential of the surrounding area, learning to integrate volcanic eruption disasters is suitable for schools whose areas have potential disasters.
20	Eddy Noviana, Otang Kurniaman, Guslinda, Munjiatun, Zufriady, Ratna Sari Dewi (2020)	disaster mitigation, disaster mitigation knowledge, forest and land fire disasters	Provide information that knowledge of forest fires and disaster mitigation land fires in elementary school students is identified as being in a low category, affecting elementary school students' age and cognitive development. Therefore, it is necessary to socialize in schools and the community through examples of disaster mitigation before, during, and after forest and land fire disasters, which are very much needed by elementary school students with cognitive level development still at the concrete operational stage.
21	W Pamungkasih, S Atun (2020)	disaster preparedness knowledge, disaster preparedness attitude, volcanic eruption	Provide information that knowledge and attitudes in dealing with Gurung Api disaster preparedness for students around Mount Merapi have good knowledge regarding knowing the danger signs of volcanic eruptions and have preparedness for disaster preparedness at a percentage of 59%.
22	Wahyu Sardjono, Harisno, Widhilaga Gia Perdana (2020)	disaster management, climate change, SECI model, knowledge management systems, dissemination	Provide information that knowledge management systems can be developed by utilizing information systems and technology to support disaster preparedness and socialization of sustainable disaster mitigation by considering the availability of good technical infrastructure, involving power holders in disaster preparedness and mitigation, having a clear organizational structure in management and government on sustainable disaster preparedness and mitigation, training using the system for all communities potentially affected by disasters, building motivation and commitment for each evaluation and implementation process.
23	Ashfaq Ahmad Shah, Zaiwu Gong, Muhammad Ali, Ruiling Sun, Syed Asif Ali Naqvi, Muhammad Arif (2020)	school children, perception, knowledge, preparedness, flood risk management/reduction	Providing information that disaster education for children in schools is one of the keys to reducing vulnerability and increasing resilience to disasters. Most of the respondents have experienced floods. Children in Nowshera know more about flood disasters, most likely due to their flood experience. Meanwhile, school-level preparedness is generally low in the research districts, and it is known that Nowshera has a better level than other districts. The level of preparedness is adequate, but the level of readiness in other district schools is still considered low.
24	Yunhao Zhang, Jun Zhu, Qing Zhu, Yakun Xie, Weilian Li, Lin Fu, Junxiao Zhang & Jianmei Tan (2020)	Landslide disaster scene, virtual disaster environment, knowledge graph, deep neural network, personalized recommendation	Provide information on several things that need to be improved in the knowledge domain. It should be studied more like knowledge usually used to construct knowledge graphs. The construction of various disasters should be studied and not limited to landslides. Then the development of technology and technical updates can be applied to construct the virtual disaster environment in the future to provide more accurate recommendations.
25	Ayu Nurul Chotimah (2019)	knowledge, attitude, landslide disaster	Increase knowledge and attitudes to improve preparedness for landslide disasters.
26	Evi Supriatun, Uswatun Insani, Arriani Indrastuti (2019)	landslide disaster, photovoice interactive, youth	Provide information that the interactive photovoice method is an innovative method that can be used in effective health education to increase knowledge and attitudes in adolescent's adolescent age group and increase knowledge and attitudes in handling landslide disasters.
27	Puspita Annaba Kamil, Sugeng Utaya, Sumarmi, Dwiyono Hari Utomo (2019)	geographic literacy, disaster knowledge, teaching material, high school	Demonstrate increased student knowledge and understanding through geographic literacy given to students on disaster-related materials.
28	Joseph Anthony L.Reyes, Kiss Alexis R.Ayo, Maria Paula M.Baluyan, Alan Sam Ralei B.Balaguer (2019)	indigenous knowledge, disaster risk reduction, natural hazards, island communities, Philippines	Provide information that local knowledge can complement and positively influence disaster management plans.
29	Pribat Rai, Vimal Khawas (2019)	traditional knowledge; indigenous knowledge; scientific knowledge; disaster risk management; disaster risk reduction	Provide information that traditional knowledge cannot be used for disaster risk reduction unless the local community recognizes and uses it daily. Local wisdom or knowledge will only become common in society without recognition and use by local communities.
30	Pei-Shan Sonia Lin, Kai-Min Chang (2019)	involution, knowledge integration, hybrid knowledge, disaster risk reduction, evacuation shelter	Provide information to formulate disaster management strategies appropriate to the local context. The capacity to solve community problems is needed to integrate scientific-based knowledge through government policies and local knowledge from the community's point of view.

In addition to discussing disaster knowledge, 30 articles that were analyzed also discussed local knowledge and wisdom related to the environment and nature in society, especially disaster mitigation knowledge. Local knowledge is the understanding, skills, and philosophy of specific communities that contain science and technology, traditional knowledge, or local wisdom. Local knowledge or local wisdom is needed in the implementation of disaster education. It is because education and culture are formed into a system that can support achieving educational goals. The following is an overview of local wisdom regarding disasters in Indonesia [15], as shown in Table 5.

**Table 5:** Classification of indigenous disaster mitigation by themes belief.

Forms of disaster mitigation	Region
*Awig-Awig* (customary rules) aims to maintain forest sustainability as a form of mitigation of landslides and weather changes	Tenganan Village, Bali
*Pikukuh* by the *Baduy* Community has forest-related rules and prohibitions aimed at avoiding soil erosion and landslides	Kanekes Village, Lebak, Banten
*Smong*, namely tsunami mitigation in the form of a story, when an earthquake occurs and is followed by sea waves, the people of Simeulue shout the word Smong to prepare to save themselves	Aceh's Simeulue Islands
*Lubuk Larangan* aims to preserve the forest by prohibiting any form of activity in riverside areas to reduce the danger of flooding and drought	Lubuk Beringin Village, Jambi
The *Minangkabau* people's sayings are *Jikok Takuik Dilamun Ombak, Jan Barumah di Tapi Pantai* (If you are afraid of waves, do not live near the beach) *Sakali Aia Gadang, Sakali Tapian Barubah* (Once the flood hits, the banks change), *Bak Aua Jo Tabiang* (like bamboo with cliffs)	West Sumatera
The Legend of the *Bakeng* Giant and the Story of the King of Kendahe. The content of this story is about the eruption of Mount *Awu*, which continues to be maintained take mitigation and evacuation actions when Mount *Awu* erupts	Sangihe Island, North Sulawesi
Folklore of the giant beetle (Banga). The experience of the Lio community in Pemo Village in responding to a tectonic earthquake, they rushed out, entered under beds or tables, hugged each other, shouted “*Epu Weo, Epu Weo, Kami Zatu, Kami Zatu, Banga Sodho tipu, Banga Tipu*” (earthquake, earthquake, we exist, we exist, a giant deceptive beetle, he deceives)	Pamo Village, Kelimutu, Ende, East Nusa Tenggara
*Sedekah Bumi* and *Gugur Gunung* are forms of tradition in preserving plants as a form of drought disaster mitigation	Segoromulyo Pamotan Village, Rembang, Central Java
The people of *Mukebuku* and *Lakamola* believe in the myth of a dragon as the cause of tectonic earthquakes, and when an earthquake occurs, they shout, “*Ami Nai Ia O*″	Mukebuku Village and Lakamola Village, East Rote, East Nusa Tenggara
Communities around Mount Kelud have a belief that the inhabitants of Mount Kelud, namely “*Lembu Suro*”, will come to meet the Elders of the Community (*Juru Kunci* of Mount Kelud) to inform them that Mount Kelud will erupt	Pandansari Village, Ngantang District, Malang Regency, East Java
The people of *Kota Pariaman* believe that a disaster will come marked by the number of birds flying by making noise, the catch of fishermen who are enough to eat or not getting results, the structure of the soil and sand near the beach which feels soft and empty when stepped on and the appearance of clouds with a straight line pattern	Pariaman, West Sumatra
*Tanda ingat ke anak cucu*, (a sign of remembrance for posterity), *merusak hutan hatinya malu* (destroying the forest he is ashamed of himself*), tanda ingat ke hari tua* (a sign of remembering the old days), *laut dijaga, bumi dipelihara* (protect the sea, protect the earth), *tanda ingat ke hari kemudian* (a sign of remembering the day after*), taat menjaga laut dan hutan* (obedient to protect the sea and the forest)	Riau

Source: adaptation of traditional disaster mitigation with the theme of belief [15].

**Fig. 2:**
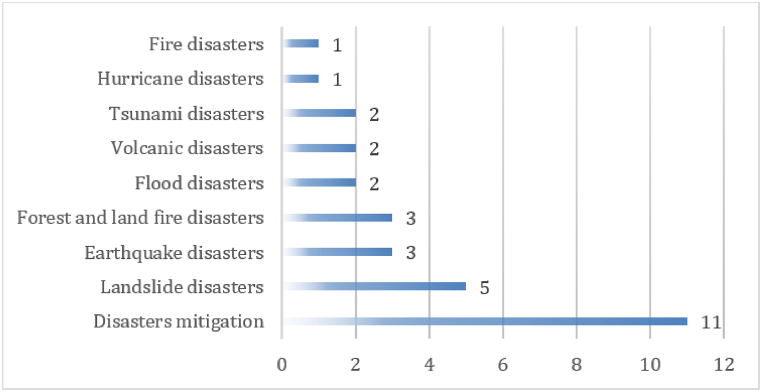
Classification of disaster knowledge.

Above all, Table 4, Table 5 describe fundamental facts regarding the source of the data, the result of research and analysis of disaster knowledge, the classification of indigenous disaster mitigation by themes belief, and the classification of disaster knowledge can be seen in Fig. 2 as follows.

Fig. 2 is a summary of 30 articles that provide information on the types of disasters explored from research on disaster knowledge, which are (a) disaster mitigation with a percentage of 36.67% or as many as 11 articles, (b) landslides with a percentage of 16.67% or as many as five articles, (c) earthquake disasters and forest and land fire disasters with a percentage of 10% each or as many as three articles, (d) floods, volcanoes and tsunamis with a percentage of 6.67% each or two articles, and (e) tornado and fire disasters with a percentage of 3.33% each or as much as one article. The data reveals that the knowledge that is most widely researched is knowledge of disaster mitigation.

### 3.2 Network analysis

#### 3.2.1 Co-occurrence network

**Fig. 3:**
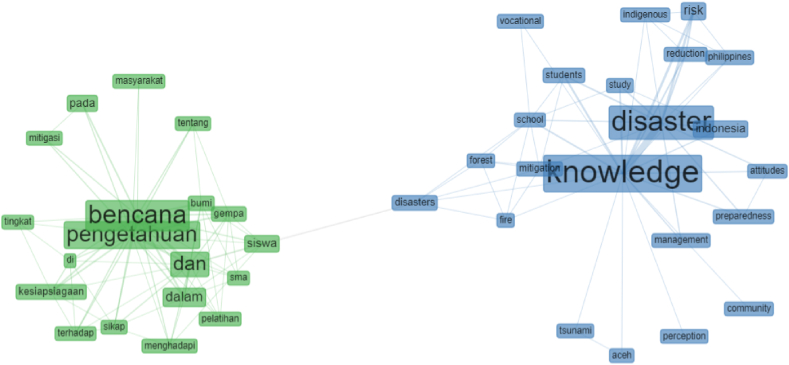
Network conceptual structure.

The conceptual structure network is the connection between the main keywords of 30 articles—the conceptual structure network of the two main keywords, disaster knowledge and disaster knowledge. The selection of the two keywords was carried out to find out connections between articles using English and Indonesian. The conceptual structure network of the two main keywords, namely “*disaster knowledge*” and “*pengetahuan bencana”*. An overview can be seen in Fig. 3.

Fig. 3 describes the conceptual structure network on the keyword “*disaster knowledge*”, which is connected to risk, education, indigenous, students, mitigation, management, perception, preparedness, attitude, community, and disasters. In addition, the keyword "*pengetahuan bencana*" has a connection with *Sikap* (attitude), *Kesiapsiagaan* (preparedness), *Mitigasi* (mitigation), *Masyarakat* (community), dan *Pelatihan* (training). It provides a close relationship between knowledge, disaster knowledge, and disaster mitigation knowledge as a necessary component of disaster mitigation education [40].

#### 3.2.2 Word cloud

**Fig. 4:**
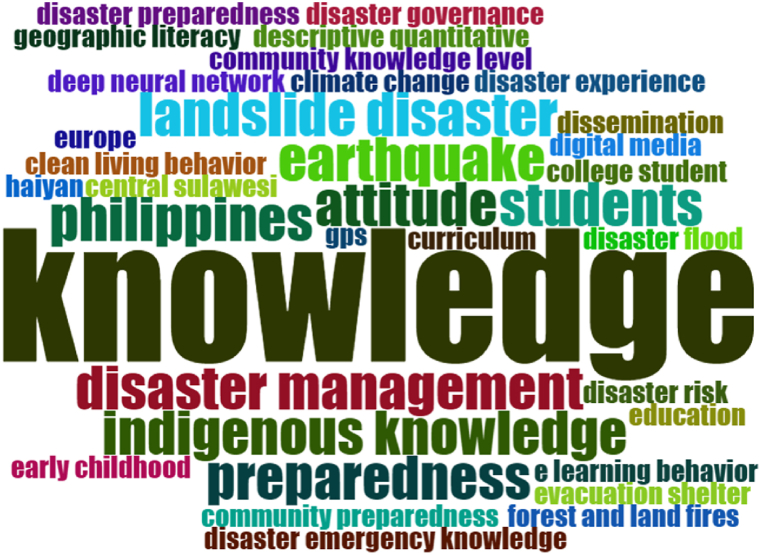
Word cloud analysis.

Based on the 30 articles, the reference sources analyzed in this research produce a list of words that are often used in articles. The list of words that are used more and more will be more extensive in size, and vice versa. Word list analysis can be seen in Fig. 4.

Based on Fig. 4, the list of words that are often used is the word “knowledge,” the word “disaster management,” the word “indigenous knowledge,” and the word “preparedness.” It indicates that knowledge has an essential component for someone to have. Knowing someone can be the basis for behaving and acting. By having local knowledge about the disaster, it is hoped that it can impact attitudes and actions to deal with disasters. Therefore, disaster education is essential because disaster education is a means to provide disaster knowledge and disaster mitigation knowledge.

#### 3.2.3 Word tree maps analysis

**Fig. 5:**
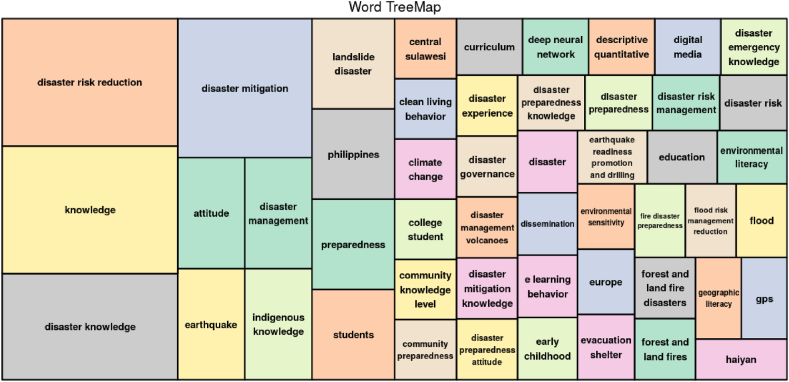
Word tree map.

30 articles that were analyzed produce a summary of the level of connectedness between concept structures. An overview can be seen in Fig. 5.

Regarding Fig. 5 description above, it is clear that disaster risk reduction, knowledge, disaster knowledge, and disaster mitigation have comparable and significant levels of connectedness. It implies that disaster risk reduction can be made through increasing knowledge, disaster knowledge, and mitigation. Knowledge, disaster knowledge, and disaster mitigation are components of disaster education [[41], [42], [43]]. Thus, disaster education can provide knowledge about disaster knowledge, and knowledge of disaster mitigation as a form of disaster risk reduction efforts [[44], [45], [46]].

#### 3.2.4 Network visualization

**Fig. 6:**
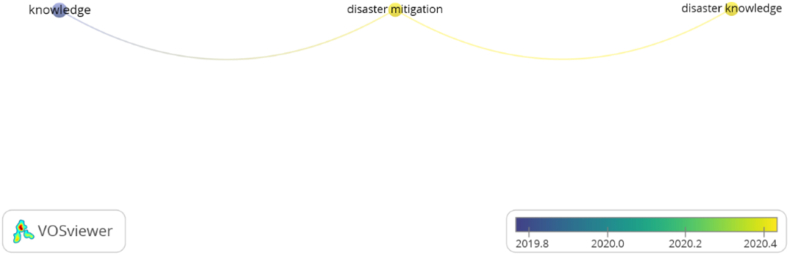
Connection visualization between concept structures.

The summary of 30 articles published from 2019 to 2022 indicates an interrelation of the concept structures. The findings from the connectedness regarding the concept structures of knowledge, disaster mitigation, and disaster mitigation knowledge, it can be shown in Fig. 6.

Fig. 6 provides information about the interrelation of concept structures. The concept structures are knowledge, disaster mitigation, and knowledge of disaster mitigation. It indicates that implementing disaster education requires knowledge, disaster mitigation, and knowledge of disaster mitigation. If one looks at the relationship between the knowledge structure and disaster mitigation, a new concept will emerge from knowledge of disaster mitigation. Clearly, in designing didactic transpositions in disaster mitigation education, components of knowledge, disaster mitigation, disaster knowledge, and disaster mitigation knowledge are needed.

## 4 Discussion

This research was conducted as a preliminary study in designing and developing didactic transposition in disaster education. Based on the research findings, four forms of connectedness were obtained and classified based on the main keyword, disaster knowledge. The four connectedness are illustrated in the form of (a) co-occurrence network analysis, (b) word cloud analysis, (c) word tree maps analysis, and (d) network visualization analysis. Furthermore, the findings of the four connectedness are grouped into four clusters. The first cluster is disaster risk reduction, the second is knowledge, the third is disaster mitigation, and the fourth is disaster knowledge. The concept structure's connectedness for each cluster can be explained as follows.

### 4.1 Cluster 1. disaster risk reduction

**Fig. 7:**
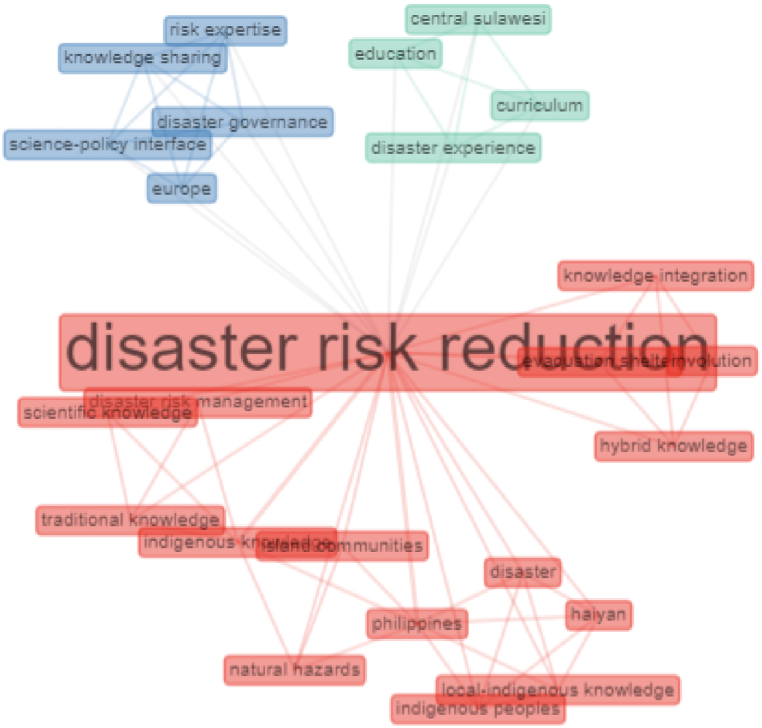
Connectivity analysis in cluster 1.

The connectedness of the concept structure to the cluster on disaster risk reduction can be seen in the red network. Disaster risk reduction is connected, among others, with the concept of knowledge integration, the concept of scientific knowledge, the concept of hybrid knowledge, the concept of disaster risk management, the concept of traditional knowledge, the concept of indigenous knowledge, and indigenous people [[47], [48], [49]]. To see the connectedness of the concept structures in the first cluster can be illustrated in Fig. 7.

Fig. 7 illustrates the relationship between disaster risk reduction and various knowledge, in this case, knowledge about the disaster. Disaster risk reduction is disaster management that emphasizes disaster management efforts on disaster risk identification in the form of vulnerabilities and hazards and developing the capacity to reduce disaster risk [2,[50], [51], [52]]. It shows that traditional knowledge, scientific knowledge, and or hybrid knowledge about disaster have essential functions and roles in reducing disaster risk [53,54].

### 4.2 Cluster 2. knowledge

**Fig. 8:**
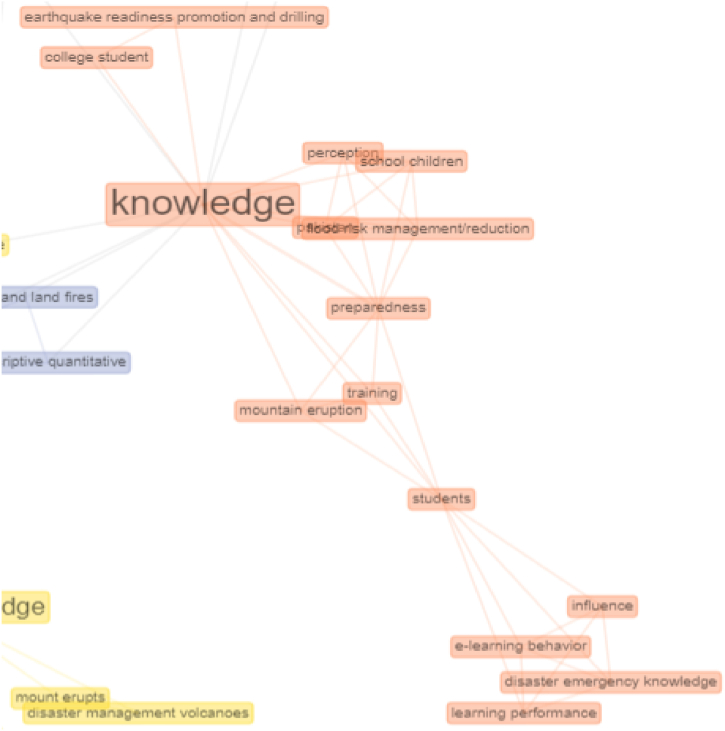
Connectivity analysis in cluster 2.

The connectedness of the concept structure to the knowledge cluster can be seen in the orange network. Knowledge is related, among others, to learning performance, e-learning behaviour, disaster emergency knowledge, preparedness, student, and perception. The connection indicates that knowledge is needed in disaster education [55,56]. The knowledge provides perceptions and attitudes, becoming actions [57,58]. With the knowledge of disaster, various information regarding the types of disasters is obtained, which are estimated disaster coverage areas, disaster symptoms, procedures for escaping, recommended places to evacuate, and other information that would significantly assist communities in dealing with disasters and reducing the impact and risks of disasters [3,59,60]. Connectivity analysis in the second cluster can be seen in Fig. 8.

### 4.3 Cluster 3. disaster mitigation

**Fig. 9:**
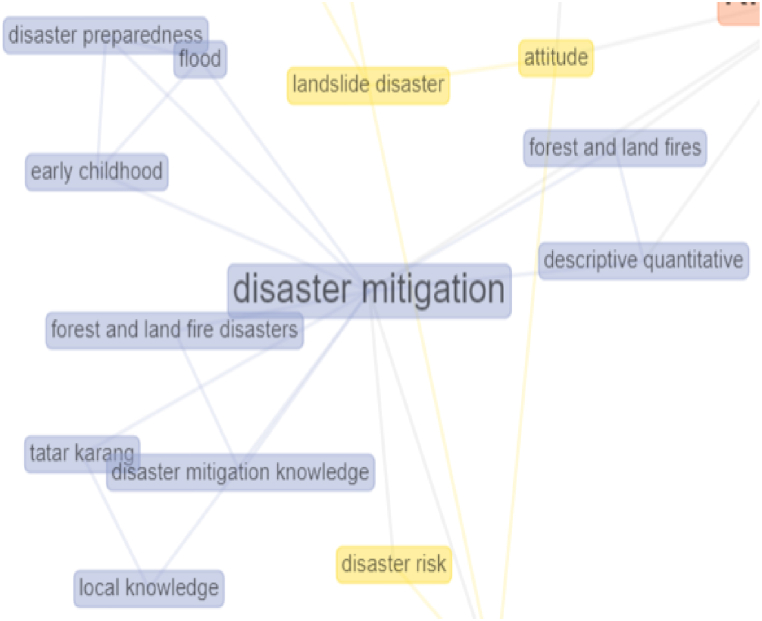
Connectivity analysis in cluster 3.

The connectedness of the concept structure in cluster three on disaster mitigation can be seen in the grey network. Disaster mitigation interconnects with disaster preparedness, local knowledge, and disaster mitigation knowledge. The connectedness regarding the concept structures in cluster one can be illustrated in Fig. 9.

Fig. 9 shows that the network of connectedness between the structures of the concept of disaster mitigation is slightly discussed in research on the disaster. The concept of disaster mitigation should be essential because disaster mitigation is an effort to reduce the risks and impacts caused by disasters on people in disaster-prone areas [[61], [62], [63]], natural disasters or man-made disasters, or a combination of both within a country or society.

### 4.4 Cluster 4. disaster knowledge

**Fig. 10:**
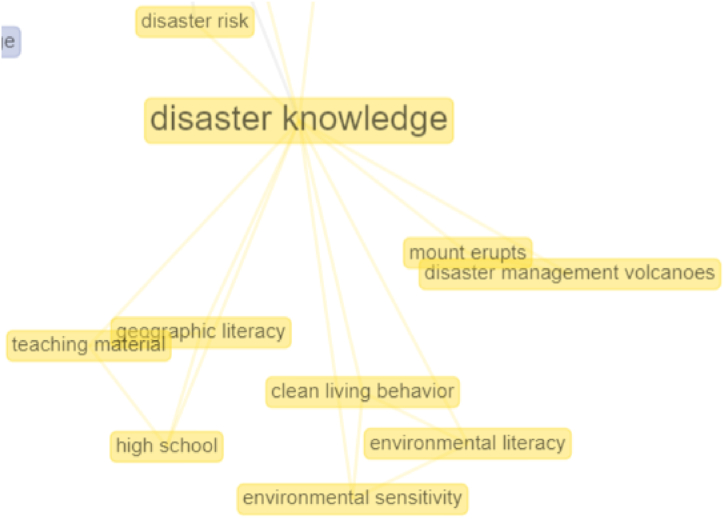
Connectivity analysis in cluster 4.

The connectedness of the concept structure to the disaster knowledge cluster can be seen in the yellow network. Disaster knowledge is connected, among others, to disaster risk and environmental literacy. The focus of studies on disaster knowledge is slightly discussed in research on disasters, even though this is very important and necessary [40,[64], [65], [66], [67]]. Disaster knowledge is an ability to remember events that disrupt people's lives, both by natural and non-natural factors and human factors that can result in casualties, environmental damage, loss of property, and psychological impacts. Connectivity analysis in cluster four can be seen in Fig. 10.

### 4.5 Connectedness of the concept structure of the four clusters

**Fig. 11:**
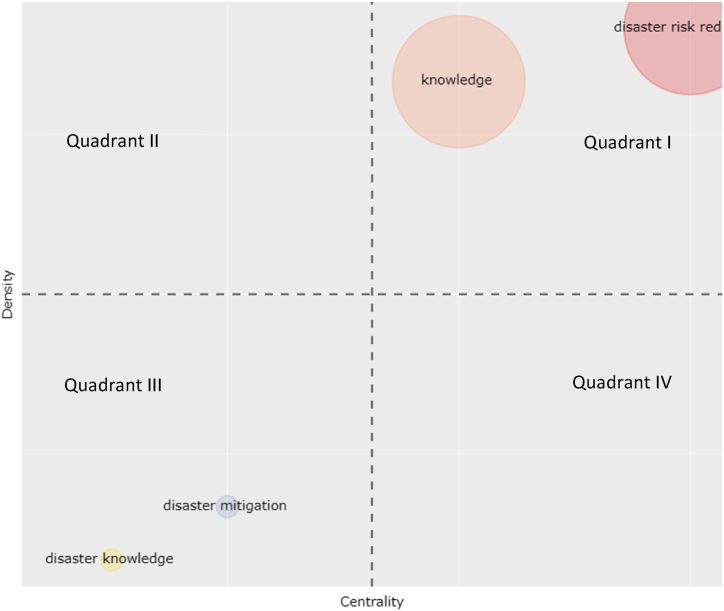
Thematic map.

The four clusters of the connectedness of the concept structure are cluster one disaster risk reduction, cluster two knowledge, cluster three disaster mitigation, and cluster four disaster knowledge. The position of each cluster can be seen in Fig. 11.

Based on Fig. 11 above, clusters one and two are in Quadrant I, while three and four are in quadrant III. Quadrant I implies that the relationship between the conceptual structure in the disaster risk reduction cluster and the knowledge cluster has a positive relationship. Moreover, Quadrant III indicates that the concept structure's connectedness to the disaster mitigation and disaster knowledge clusters has a negative relationship.

A positive relationship in the Quadrant I means that disaster risk reduction and knowledge are the concepts most widely discussed and used as the focus of studies in research on disaster education. Meanwhile, the negative connectedness in Quadrant III indicates that disaster mitigation and disaster knowledge are the concepts that are slightly discussed and are the focus of discussion and study in research on disaster education. Knowledge of disaster and disaster mitigation is essential to ensure the availability and accessibility of accurate and reliable disaster risk information [10,[68], [69], [70]] through effective learning and disaster education implementation [19,71]. Disaster education can identify key factors, knowledge of disaster, and knowledge of mitigation [40] that will be driving the success of disaster management [10,72,73] so that the focus of studies on disaster mitigation and disaster knowledge [[74], [75], [76], [77]] is used as a basis as preliminary study in the design and development of didactic transpositions in disaster education.

## 5 Conclusion

Preliminary research has been described in this research to show the didactic transposition in disaster education. Based on the bibliometric analysis, four clusters were disaster risk reduction, knowledge, disaster mitigation, and disaster knowledge. The position of the disaster risk reduction and knowledge cluster was in Quadrant I, while the disaster mitigation and disaster knowledge cluster was in Quadrant III. Quadrant III was the position that slightly discussed the study of disaster education research. Therefore, it is recommended for future research to make design and development of didactic transpositions in disaster education for prospective elementary school teachers. In addition, it can be utilized and used in learning about disaster content in elementary schools. It is expected that the disaster mitigation knowledge possessed by prospective teachers in elementary schools can be transferred back to students so that students can have disaster knowledge and disaster mitigation knowledge, and learn resilience in dealing with disasters.

## Author contribution statement

All authors listed have significantly contributed to the development and the writing of this article.

## Funding statement

This research did not receive any specific grant from funding agencies in the public, commercial, or not-for-profit sectors.

## Data availability statement

Data will be made available on request.

## Declaration of competing interest

Dominikus David Biondi Situmorang has a position to declare that he is one of the members of the journal's Editorial Teams or Associate Editor. However, this article is handled by another unknown Associate Editor and reviewed by the Reviewers objectively and double-blind, based on applicable regulations from Elsevier, Heliyon, and Cell Press.
