# Corrigendum to “Why is didactic transposition in disaster education needed by prospective elementary school teachers?” [Heliyon 9(4) (April 2023) e15413]

**DOI:** 10.1016/j.heliyon.2023.e16575

**Published:** 2023-05-24

**Authors:** Eddy Noviana, Almasdi Syahza, Zetra Hainul Putra, Hadriana, Yustina, Sri Erlinda, Desfi Rahmi Putri, M. Arli Rusandi, Dominikus David Biondi Situmorang

**Affiliations:** a Doctorate Program of Education, Universitas Riau, Indonesia; b LPPMP, Universitas Riau, Indonesia; c Department of Guidance and Counseling, Universitas Riau, Indonesia; d Department of Guidance and Counseling, Atma Jaya Catholic University of Indonesia, Indonesia

In the original published version of this article, the authors incorrectly added “Department of Guidance and Counseling, Atma Jaya Catholic University of Indonesia, Indonesia” as an affiliation for Eddy Noviana and Zetra Hainul Putra. The affiliations have now been corrected. The authors apologize for the error. Both the HTML and PDF versions of the article have been updated to correct the errors.

